# Prevalence of schistosome infection among children under two years of age: a brief report from medium-to-high endemic regions of *Schistosoma mansoni* in Madagascar

**DOI:** 10.1186/s41182-025-00871-w

**Published:** 2025-12-04

**Authors:** Irina Kislaya, Rivo Andry Rakotoarivelo, Tahimandranto Rasamoelina, Jeannine Solonirina, André Brito, Elveric Fesia Ratiaharison, Ravo Razafindrakoto, Nantenaina Matthieu Razafindralava, Njary Rakotozandrindrainy, Mickael Radomanana, Mala Rakoto Andrianarivelo, Philipp Klein, Anna Jaeger, Eva Lorenz, Jule Hameister, Pytsje T. Hoekstra, Paul L. A. M. Corstjens, Norbert Georg Schwarz, Govert J. van Dam, Jürgen May, Valentina Marchese, Raphäel Rakotozandrindrainy, Daniela Fusco

**Affiliations:** 1 Implementation Research, Bernhard Nocht Institute for Tropical Medicine, Hamburg, Germany; 2 German Center for Infection Research, (DZIF), Hamburg-Borstel-Lübeck-Riems, Germany; 3 Department of Infectious Diseases Epidemiology, Bernhard Nocht Institute for Tropical Medicine (BNITM), Hamburg, Germany; 4 Association Promotion de la Santé, de l’intelligence artificielle et du Numérique (APSIAN), Fianarantsoa, Madagascar; 5 Infectious Diseases Service, University Hospital Tambohobe, Fianarantsoa, Madagascar; 6 Centre d’Infectiolologie Charles Mérieux (CICM), Antananarivo, Madagascar; 7 University Antananarivo, Antananarivo, Madagascar; 8 Center for Infectious Diseases, Leiden University, Leiden University Medical Center, Leiden, the Netherlands; 9 Dept. Cell and Chemical Biology, Leiden University Medical Center, Leiden, the Netherlands

**Keywords:** Schistosomiasis, Madagascar, Prevalence, Children under 2 years of age

## Abstract

**Introduction:**

Schistosome infections in early childhood can affect a child’s growth and development, leading to lifelong consequences. Historically, monitoring and control of schistosomiasis have focused primarily on school-aged children leading to a knowledge gap on the magnitude of the prevalence of infections in younger age groups in many endemic countries. This study aimed to estimate the prevalence of schistosome infections among children under 2 years of age and describe its distribution in three regions of Madagascar endemic for *Schistosoma mansoni*, thereby informing public health strategies in the country.

**Methods:**

A cross-sectional study was conducted on a sample of 2018 children under 2 years of age recruited from 42 primary health care centres in the regions of Itasy, Bongolava and Amoron’i Mania, Madagascar, from March 2020 to June 2021. Urine samples were collected to perform an up-converting reporter particle lateral flow circulating anodic antigen assay (UCP-LF-CAA) for the detection of schistosome infections. To identify factors associated with the prevalence of schistosome infection, prevalence ratios with 95% CIs were estimated using mixed-effects Poisson regression.

**Results:**

Among 2018 sampled children with an average age of 9.6 months (SD = 1.2), the prevalence of schistosome infection was 6.2% [CI95%: 5.0, 7.8]. The prevalence estimates were similar across all population subgroups. We observed no statistically significant associations of schistosome infections in children with maternal age (*p*-value = 0.4110), education (*p*-value = 0.1281), occupation (*p*-value = 0.3333), child sex (*p*-value = 0.3692), urbanisation (*p*-value = 0.8272) or region of residence (*p*-value = 0.7425).

**Conclusion:**

Our results show that the prevalence of schistosome infection in children under 2 years of age in Madagascar is significant. Given the high burden and long-term consequences of early schistosome infection, integrated and inclusive public health interventions that combine treatment, caregiver health education, with improvements in sanitation and access to clean water, are needed for children under 2 years of age in endemic settings.

## Background

Schistosomiasis is a neglected disease of poverty, estimated to affect 253.4 million people across 78 countries worldwide, with a particularly high burden in children, affecting more than 135.5 million of them [1]. Infections during early childhood can cause impaired growth, anaemia, and delays in cognitive development, resulting in irreversible long-term consequences that contribute to perpetuating the vicious cycle of poverty [2, 3]. Moreover, schistosome infections can significantly impair immune responses to vaccines and accelerate the waning of vaccine-induced immunity [4, 5], leaving children vulnerable to other infectious diseases.

Preventive chemotherapy with praziquantel (PZQ) through Mass Drug Administration (MDA) campaigns represents the primary public health strategy for controlling the disease in endemic countries [6]. Historically, both surveillance and control of schistosomiasis through MDAs mainly targeted school-aged children, neglecting other vulnerable groups such as those under 2 years of age. However, a shift in global health strategies from control to elimination of schistosomiasis as a public health problem has encouraged the expansion of MDAs to other vulnerable at-risk groups including pre-school-age children (PSAC) [6]. The most recent guidelines from the World Health Organization (WHO) recommend MDAs annually in settings with a schistosomiasis prevalence of 10% or higher and also advise complementing the school-based approach with community-based MDAs, and target the entire population, including children aged 2 years in highly endemic settings [6]. For children under 2 years of age, current guidelines recommend a test-and-treat approach, based on clinical judgement. However, limited healthcare access in many endemic countries, along with diagnostic and operational challenges related to sample collection, availability of point-of-care tests and PZQ administration, hinders adherence to these guidelines in clinical practice [7, 8].

Globally, it is well established that PSACs are susceptible to schistosomiasis infection, particularly in high-transmission areas [9, 10]. Among the 47 countries in the WHO African region, 24 had at least one published study on schistosomiasis among PSAC, reporting highly heterogeneous prevalence estimates, in the range from 1 to 82% [1, 9, 10]. However, most of these studies are region-specific rather than nationwide, are based on a limited sample size and overlook children younger than 2 years [9, 10].

Madagascar is a country where schistosomiasis occurs with a high burden, with 106 out of 113 districts considered endemic, and a prevalence of disease exceeding 50% in 40.7% (*n* = 46) of the districts [11]**.** Several epidemiological studies have reported schistosomiasis prevalence above 50% among the general population [12] and pregnant women in Madagascar [13], suggesting a high likelihood of early infections in PSAC. To date, in this country, there is a knowledge gap regarding the prevalence of schistosome infection in PSAC, with only one pilot study based on 89 participants in 2- to 4-year-olds in Marolambo District [14], and no studies or data available for children under 2 years of age yet.

This study aims to present estimates of schistosome infection prevalence in children under 2 years of age and to identify associated factors, addressing existing research gaps and raising attention to this issue at both national and global scales.

## Methods

### Study settings

This was a cross-sectional study that used data from an existing cluster randomised controlled clinical trial project called freeBILy (fast and reliable easy-to-use-diagnostics for eliminating bilharzia in young children and mothers) [15]. Data for the present study were collected between March 2020 and June 2021 in 42 primary health care centres in the regions of Itasy, Bongolava and Amoron’i Mania of Madagascar, known to be endemic for *Schistosoma mansoni* [16]. Children of mothers participating in the freeBILy trial (Pan-African Clinical Trial Register PACTR201905784271304) were enrolled during one of the follow-up visits at 9 months of age [15].

### Data collection and laboratory testing

Demographic data and urine samples were collected by trained nurses or midwives. The up-converting reporter particle lateral flow circulating anodic antigen test detecting circulating anodic antigen (UCP-LF CAA) was used for the detection of the *Schistosoma* genus-specific CAA antigen as a marker of active infections, as described previously [17], using a 2 pg/ml cut-off for positivity (UCAA*hT*417). This assay has previously shown high specificity and sensitivity in detecting four *Schistosoma* species (*Schistosoma mansoni, Schistosoma haematobium, Schistosoma japonicum and Schistosoma mekongi*) in regions with different endemicity settings [17, 18].

### Statistical analysis

The prevalence of schistosome infection was estimated for the overall sample and stratified by child (sex, region of residence, urbanisation) and maternal characteristics, such as education level, occupation as a farmer, and treatment with PZQ during pregnancy. To identify factors associated with the prevalence of schistosome infection, we used a mixed-effects Poisson regression to calculate prevalence ratios (cPR) and 95% confidence intervals (CI), accounting for the hierarchical data structure.

## Results

### Participants’ characteristics

**Fig. 1:**
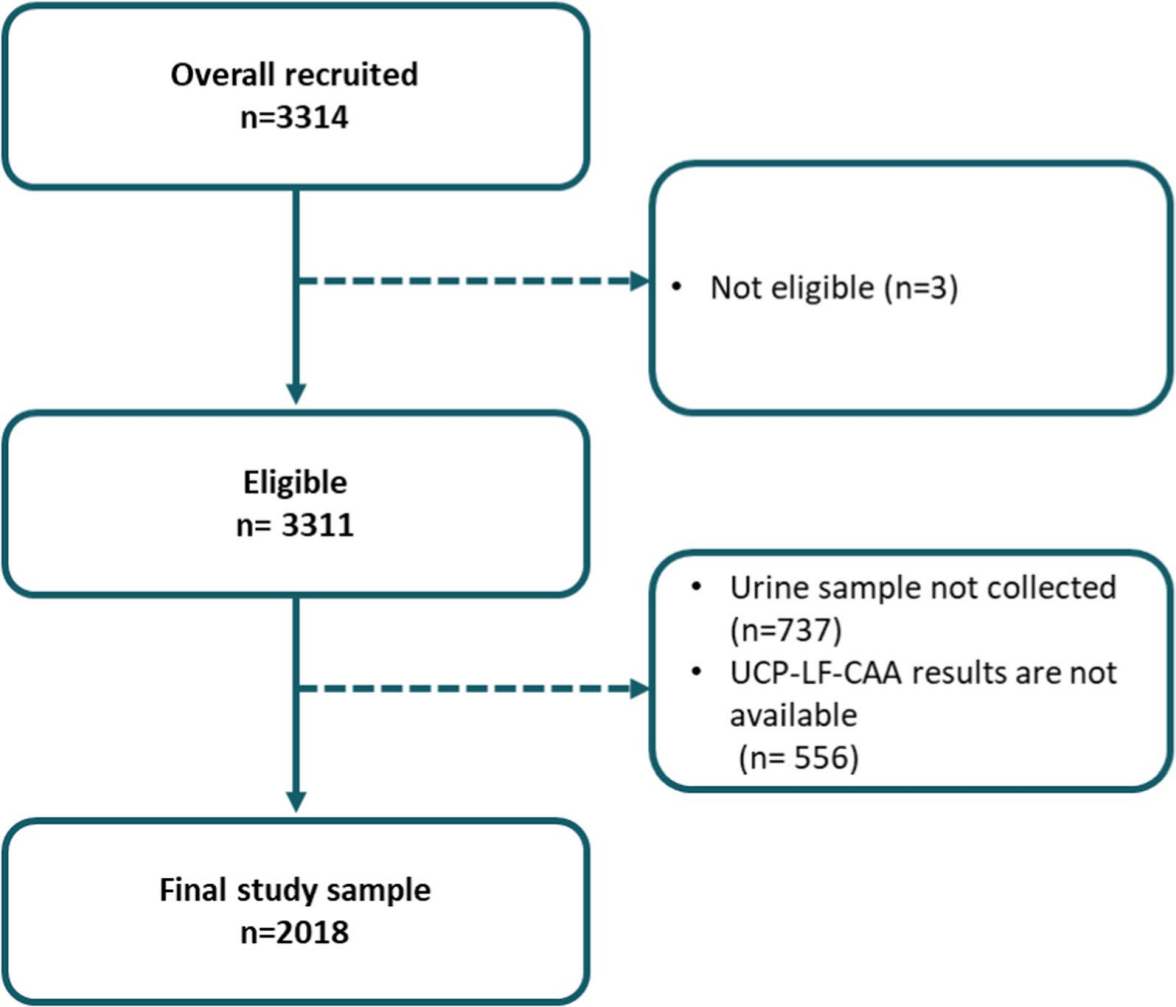
Participants exclusion flowchart

Of the 3311 eligible participants, 2018 were included in the study (Fig. 1).

The average age of the participants was 9.6 months (SD = 1.2). Among them, 23.7% were from urban areas and 71.1% belonged to families where the primary mother’s occupation was farming (Table 1).

**Table 1:** Characteristics of children and mothers

	n	%, mean (SD)
Urbanisation		
Rural	1540	76.3
Urban	478	23.7
Region		
Amoron’i Mania	978	48.5
Bongolava	230	11.4
Itasy	810	40.1
Sex of child		
Male	1082	53.6
Female	936	46.4
Age, months*	2018	9.6 (1.2)
Weight, kg*	2018	8.0 (1.0)
Length, cm*	2016	69.0 (3.4)
Chief of family		
Mother	67	3.3
Other	1951	96.7
Mother’s occupation		
Farming	1434	71.1
Other	584	28.9
Mother’s education		
Never went to school	75	3.7
Primary school	1055	52.3
Secondary school or higher	888	44.0
Mother’s age group		
16–20 years	522	25.9
21–25 years	636	31.5
26–30 years	447	22.2
31 + years	413	20.5
PZQ treatment during pregnancy		
No	1244	61.7
Yes	774	38.4

*For numerical variables mean and standard deviation (SD) are reported

### Prevalence of schistosome infection

**Fig. 2:**
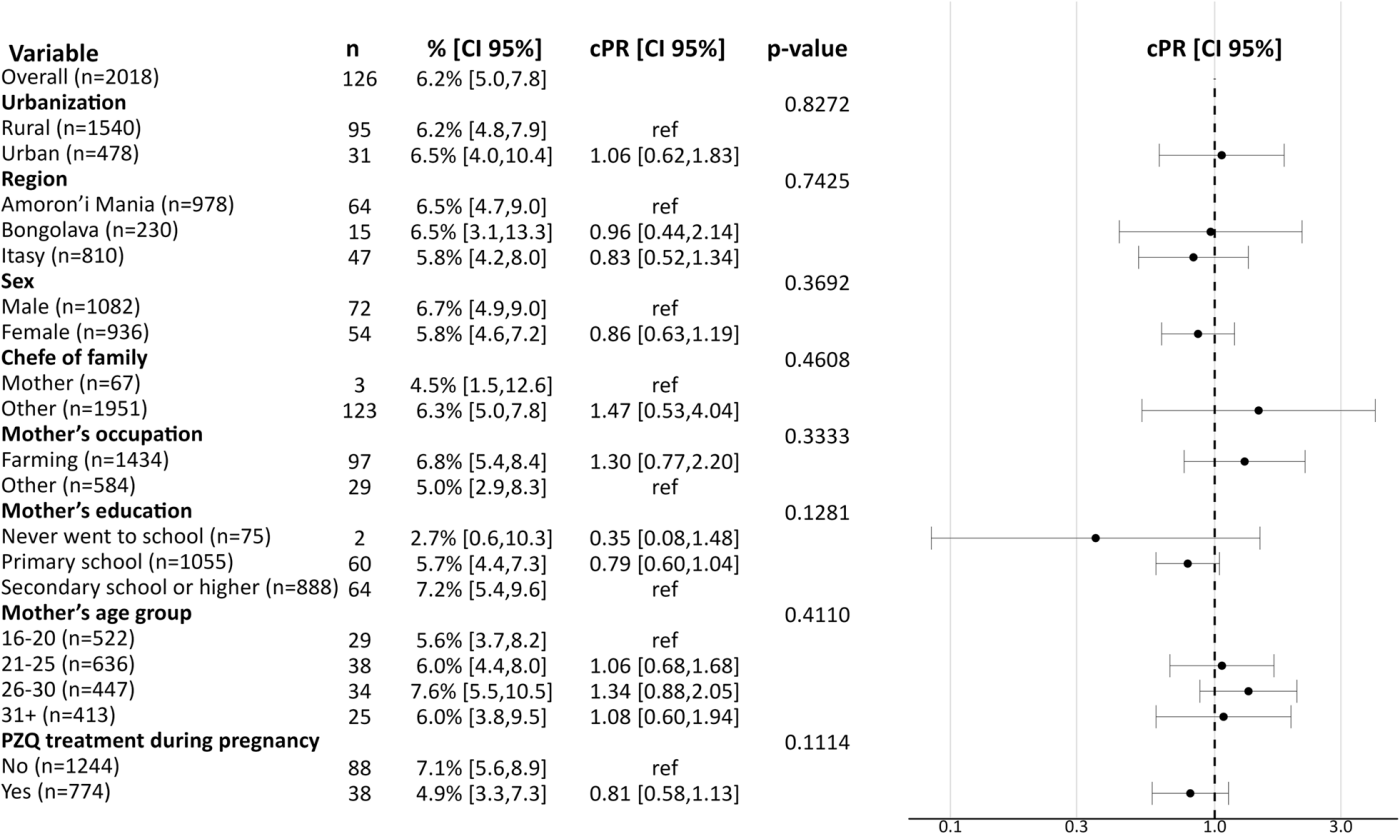
Estimates of the prevalence of schistosome infection and crude prevalence ratios (cPR) across population subgroups

Overall, 6.2% [CI95%: 5.0, 7.8] of participants tested positive for schistosome infection. The prevalence of schistosome infection was similar between boys (6.7%, [CI95%: 4.9, 9.0]) and girls (5.8%, [CI95%: 4.6, 7.3]), across regions (6.5% [CI95%: 5.4, 8.4] Amoron’i Mania, 6.5% [CI95%: 5.4, 8.4] Bongolava, 5.8% [CI95%: 5.4, 8.4] Itasy), and between rural and urban settings (6.2%[CI95%: 5.4, 8.4] vs. 6.5%[CI95%: 5.4, 8.4]) (Fig. 2).

The overall prevalence of schistosome infection among participants from families where the mother’s occupation was farming was 6.8% [CI95%: 5.4, 8.4] compared to 5.0% [CI95%: 2.9, 8.3] for other professions. Although the prevalence of infection ranged by maternal education from 2.7% [CI95%: 0.6, 10.3] in the low education group to 7.2% [CI95%: 5.4, 9.6] in the higher education group, the difference between them was not statistically significant (Fig. 2). Moreover, we observed no significant differences in prevalence rates related to maternal treatment with PZQ during pregnancy, 4.9% [CI 95%: 3.3, 7.3] in the treated and 7.1% [CI 95%: 5.6, 8.9] in the untreated group (cPR = 0.81, CI 95%: 0.58, 1.13). None of the examined factors demonstrated a statistically significant association with the prevalence of schistosome infection in a bivariate analysis (Fig. 2).

## Discussion

This large-scale cross-sectional study, conducted across 42 primary healthcare facilities using the reliable, broadly accepted UCP-LF CAA diagnostic test, addresses a knowledge gap regarding the prevalence of schistosome infection among children under 2 years of age in Madagascar. Our results show a prevalence of 6.2% in this age group. According to population projections, more than a million babies are born in Madagascar each year [19]. Extrapolating from our regional estimates, this will imply a substantial number of infected children nationally while their needs for treatment have been overlooked.

Current guidelines recommend a test-and-treat approach for children under 2 years of age [6]. The prequalification of the paediatric formulation of PZQ by the WHO in 2024 is a promising development that could bridge the treatment gap for young children and promote the implementation of MDA involving PSAC in high-transmission areas [20]**.** However, there is currently no clear global distribution strategy for paediatric PZQ. No plans for donations of the new formulation have been established yet [20], requiring countries like Madagascar to rely on their internal financial resources to provide treatment for young children. As this age group in Madagascar is not actively targeted by any organised national programs, access to treatment in primary healthcare requires out-of-pocket payments, creating strong financial constraints. Consequently, this vulnerable age group in limited resource settings will remain untreated until they become eligible for MDA. Such delays in the first treatment and accumulated exposure to infection during a critical stage of physical and cognitive development can result in irreversible, debilitating long-term consequences of infection in adulthood [21].

Integrating treatment for schistosomiasis into ongoing routine healthcare activities at the primary level of care could be a path to addressing the treatment needs of infected individuals, contributing to the improvement of the overall health of young children. Although not receiving much emphasis in recent years, successful examples of integration from Burundi, Mali, and Senegal are well known [22]. Routine vaccination visits or community-based Integrated Management of Childhood Illness programs that have been implemented in Madagascar since 2011 [23] could be leveraged for this purpose.

Implementation of a test-and-treat approach also faces diagnostic challenges. The UCP-LF CAA test used in this study, while it is highly sensitive, is primarily employed in research settings as it requires laboratory infrastructure, and has high costs relative to locally available resources. This highlights a pressing need for sensitive and affordable point-of-care diagnostic tests for routine diagnostics of schistosome infections and control programmes in limited resource settings.

This study also assessed the association between schistosome infection in young children and various sociodemographic and contextual factors, aiming to identify population subgroups that are most at risk. Our results showed no statistically significant associations with any of the factors analysed. As such, we were unable to identify specific groups which can be prioritised for intervention. This result also suggests that other exposure variables such as maternal awareness of schistosomiasis, knowledge of its transmission routes and exposure to different water sources should be explored in future studies.

This study has several limitations. First, recruiting participants from primary healthcare facilities may introduce selection bias favouring those with better healthcare access. Second, the diagnostic tool used does not enable direct identification of Schistosoma species, which in our study were inferred based on epidemiological context.

## Conclusions

Our study addresses a relevant knowledge gap regarding the prevalence of schistosome infection among children under 2 years of age in the Itasy, Bongolava and Amoron’i Mania regions of Madagascar. Although implementing studies in this age group poses several operational challenges related to biological samples collection and testing, the research community has a crucial role in informing decision-makers and supporting the implementation of control and prevention programs at the national level.

By regularly collecting valid and reliable data on disease prevalence, the safety of PZQ and its new paediatric formulation, and developing implementation studies on strategies for schistosomiasis control in PSAC, the research could help pave the way for more inclusive prevention and treatment programs.

## Data Availability

The datasets analysed during the current study are available from the corresponding author on reasonable request.

## Abbreviations

cPR: Crude prevalence ratio
CI: Confidence interval
MDA: Mass Drug Administration
PSAC: Pre-school-age children
PZQ: Praziquantel
UCP-LF CAA: Up-converting reporter particle lateral flow circulating anodic antigen
WHO: World Health Organization
